# Metabolic Determinants of Systemic Inflammation Dynamics During Hemodialysis: Insights from the Systemic Immune–Inflammation Index in a Single-Center Observational Study

**DOI:** 10.3390/metabo15100651

**Published:** 2025-09-30

**Authors:** Martina Mancinelli, Federica Moscucci, Vincenza Cofini, Anna Luisa De Nino, Raffaella Bocale, Carmine Savoia, Francesco Baratta, Giovambattista Desideri

**Affiliations:** 1 Department of Anatomical Sciences, Histological, Legal Medical and Locomotor, Sapienza University of Rome, 00161 Rome, Italy; martina.mancinelli@uniroma1.it; 2 Geriatric Unit, Department of Internal Medicine and Medical Specialties, AOU Policlinico Umberto I, 00161 Rome, Italy; federica.moscucci@uniroma1.it (F.M.); francesco.baratta@uniroma1.it (F.B.); 3 Department of Life, Health and Environmental Sciences, University of L’Aquila, 67100 L’Aquila, Italy; vincenza.cofini@univaq.it; 4 Dialysis Unit, Avezzano Hospital, 67051 Avezzano, Italy; adenino@asl1abruzzo.it; 5 Unit of Endocrinology, Department of Translational Medicine and Surgery, Università Cattolica del Sacro Cuore, Fondazione Policlinico “A. Gemelli” IRCCS, 00168 Rome, Italy; raffaella.bocale@policlinicogemelli.it; 6 Clinical and Molecular Medicine Department, Faculty of Medicine and Psychology, Sant’Andrea Hospital, Sapienza University of Rome, 00189 Rome, Italy; carmine.savoia@uniroma1.it; 7 Department of Medical and Cardiovascular Sciences, Sapienza University of Rome, 00161 Rome, Italy

**Keywords:** systemic immune-inflammation index, end stage renal disease, hemodialysis, metabolic profile

## Abstract

**Background/Objective:** Systemic inflammation is a hallmark of end-stage renal disease (ESRD) and contributes to the high burden of cardiovascular morbidity and mortality in hemodialysis (HD) patients. The systemic immune–inflammation index (SII), derived from peripheral neutrophil, lymphocyte, and platelet counts, has emerged as a promising biomarker of immune–inflammatory status. This study aimed to assess the acute effect of a single HD session on systemic inflammation and to identify metabolic predictors associated with this response. **Methods:** In this single-center observational before–after study, 44 chronic HD patients were enrolled. Blood samples were collected immediately before and after a single HD session. SII was calculated as platelet count × neutrophil count/lymphocyte count. Subgroup analyses were conducted based on renal disease etiology and diabetic status. Multivariable linear regression models identified baseline predictors of SII variation. **Results:** Median SII significantly decreased post-HD in the overall cohort (from 553.4 [342.6–847.5] to 449.1 [342.6–866.6], *p* = 0.001), with a more pronounced reduction in patients with cardiometabolic etiologies (from 643.4 [353.3–1360.0] to 539.1 [324.8–1083.4], *p* = 0.007) and diabetes (from 671.1 [408.7–1469.1] to 458.3 [285.7–1184.4], *p* = 0.028), but not in those with nephroangiosclerosis (*p* = 0.182). Baseline total cholesterol (*p* = 0.001) and gamma-glutamyl transferase (*p* = 0.034) were positively associated with smaller reductions in SII, while higher baseline glycaemia predicted a greater decrease in post-dialysis SII (*p* = 0.021). **Conclusions:** HD acutely modulates systemic inflammation, as reflected by reduction in SII. The magnitude of this response is significantly influenced by individual metabolic profiles. These findings highlight the relevance of metabolic–immune crosstalk in ESRD and suggest that SII may serve as a dynamic biomarker integrating inflammatory and metabolic signals, deserving further validation in larger, outcome-driven studies.

## 1. Introduction

Systemic inflammation and metabolic dysregulation are deeply interconnected processes that play a central role in the pathophysiology of chronic diseases, particularly in patients with end-stage renal disease (ESRD) [1,2]. Chronic systemic inflammation is a key pathophysiological mechanism underlying the progression and poor prognosis of numerous non-communicable diseases, including cardiovascular diseases (CVD) [3,4], type 2 diabetes [5,6], neurodegeneration [7], and chronic kidney disease (CKD) [8,9]. Inflammation is no longer seen merely as a defensive reaction to pathogens or tissue injury, but rather as a dynamic, systemic process that, when dysregulated, contributes to cellular dysfunction, endothelial damage, and metabolically driven immune alterations [10,11].

Patients with ESRD undergoing HD experience a state of chronic low-grade inflammation that has been strongly associated with increased cardiovascular morbidity and mortality [12,13,14], protein–energy wasting, and reduced quality of life [15,16,17]. This inflammatory state results from a combination of uremic toxins, oxidative stress, comorbidities, impaired immune responses, and the HD procedure itself [18] and might be favored by metabolic factors—such as lipid profile, glycemic control, and hepatic function [15,19]. Chronic exposure to dialysis membranes, dialysate contaminants, vascular access procedures, and contact between blood and artificial extracorporeal circuits activate innate immune pathways, promote cytokine release, and alter leukocyte behavior [15,19]. However, data on the short-term behavior of SII in response to HD and on the potential metabolic determinants of this response remain limited.

Among the various biomarkers and indexes of systemic inflammation used in clinical and research settings, the systemic immune–inflammation index (SII) has recently emerged as a novel, accessible, and cost-effective indicator of immune–inflammatory status [20,21,22,23,24,25,26]. SII is calculated using the peripheral platelet count x neutrophil count/lymphocyte count, integrating the pro-inflammatory activity of neutrophils and platelets with the regulatory and anti-inflammatory potential of lymphocytes. It has demonstrated prognostic value in solid tumors, sepsis, and cardiovascular events [20].

This study aimed to evaluate the acute effect of a single HD session on systemic inflammation, as assessed by SII, and to investigate whether baseline metabolic parameters predict the degree of inflammatory modulation. This approach offers insight into the metabolic–immune crosstalk in the HD population and may support the development of personalized dialysis strategies.

## 2. Materials and Methods

### 2.1. The Study Design and Population

This was a single-center, observational, before–after study conducted on consecutive patients referring to the Hemodialysis Unit of the SS. Filippo e Nicola Hospital in Avezzano, Italy, part of the Local Health Authority 1 (ASL1) of Abruzzo. The study protocol was approved by the Internal Review Board of the University of L’Aquila (approval no. 45/2021) and conducted in accordance with the Declaration of Helsinki. Written informed consent was obtained from all participants. Eligible patients were adults (>18 years) with ESRD undergoing chronic thrice-weekly hemodialysis. Inclusion criteria required the ability to provide informed consent and participation in a scheduled dialysis session during the data collection period in August 2023, on a stable dialysis regimen for at least 3 months. Exclusion criteria were (1) active cancer; (2) recent history (<3 months) of acute vascular events; (3) active infection; (4) recent history of hospitalization (<3 months); (5) ongoing use of immunosuppressive drugs; (6) age ≥90 years. A total of 44 consecutive patients were enrolled during morning and afternoon dialysis shifts. All patients were treated in an outpatient setting using standard 4 h HD sessions. Two types of HD machines were employed: the Fresenius Medical Care 5008 Hemodialysis System and the Flexya Hemodialysis System. Dialyzers were selected according to individual patient characteristics and included high-flux synthetic filters. Anticoagulation was performed with enoxaparin sodium, administered into the arterial line at the beginning of the session, with dosage adjusted for body weight and dialysis duration. Vascular access consisted of either native arteriovenous fistula or tunneled central venous catheter, depending on clinical indication. Dialysate composition and conductivity were standard and based on institutional protocols. Venous blood samples were collected from each patient in fasting condition immediately before and 10 min after the dialysis session.

### 2.2. Laboratory Measurements

Samples were analyzed at the hospital’s central laboratory. Complete blood counts were obtained using an automated hematology analyzer, and the following parameters were recorded: platelet count, neutrophil count, and lymphocyte count. The SII was calculated using the formula: SII = platelet count × neutrophil count/lymphocyte count. The SII relative change was calculated as “(after HD SII − baseline SII)/basal SII × 100”. Additional clinical and biochemical parameters—including serum creatinine, urea, fasting glycemia, cholesterol, triglycerides, liver enzymes (AST, ALT, GGT), and blood pressure—were also collected.

### 2.3. Statistical Analysis

Continuous variables were expressed as means ± standard deviation or medians with interquartile range, depending on their distribution. The Kolmogorov–Smirnov test was used to assess normality. Differences were tested with t-test and Mann–Whitney, respectively. Counts were expressed as number, percentage and differences tested with chi-square test. Spearman test was performed to investigate univariate correlation between baseline characteristics. Paired comparisons of pre- and post-dialysis parameters were performed using Wilcoxon signed-rank test.

Subgroup analyses were conducted according to primary renal disease etiology (cardiometabolic vs. non-cardiometabolic). Patients with primary nephroangiosclerosis and diabetic nephropathy were included in the subgroup of “cardiometabolic etiologies”, other conditions were included in “non-cardiometabolic etiologies” subgroup (etiologies are listed in detail in Supplementary Table S1). Time (before–after HD) and etiologies effects were tested using generalized linear mixed model, including sex, age >65, and the interaction time*etiologies effect. We fitted generalized linear mixed models with a random effect for patients and random slope for time at the patient level, allowing each patient to have an individual baseline and trajectory over time. A single exploratory sub-analysis, with the same model applied for the main outcome, was conducted in cardiometabolic patient’s subgroup to test differences between patients with nephroangiosclerosis and diabetic nephropathy. Due to the exploratory nature of the sub-analysis, correction for multiple tests was not performed. Multivariable generalized linear model was used to identify baseline predictors of percentage change in SII values. Model included age, sex, cardiometabolic etiologies, total cholesterol, GGT, glycaemia, mean blood pressure, basal SII, and time in HD. A two-tailed *p*-value < 0.05 was considered statistically significant. All analyses were performed using SPSS Statistics v28 (IBM Corp., Armonk, NY, USA).

## 3. Results

A total of 44 patients with ESRD were enrolled. Among them, 20 patients (45.5%) had cardiometabolic conditions as the primary cause of renal failure. Table 1 presents the baseline characteristics of the entire cohort and is stratified by etiology and the etiologies that led to ESRD. Patients with cardiometabolic etiology were significantly older (*p* = 0.031) and had a higher prevalence of diabetes (*p* = 0.003) and hypertension (*p* = 0.004).

Baseline median SII was 553.4 [342.6–847.5] in the overall population. A trend toward higher median values was observed in patients with cardiometabolic etiologies (643.4 [353.3–1360.0]) compared to those with non-cardiometabolic etiologies (483.2 [337.4–630.8]); however, this difference did not reach statistical significance (*p* = 0.109). No significant univariate correlations were found between baseline characteristics and SII, as detailed in Supplementary Table S1.

**Figure 1:**
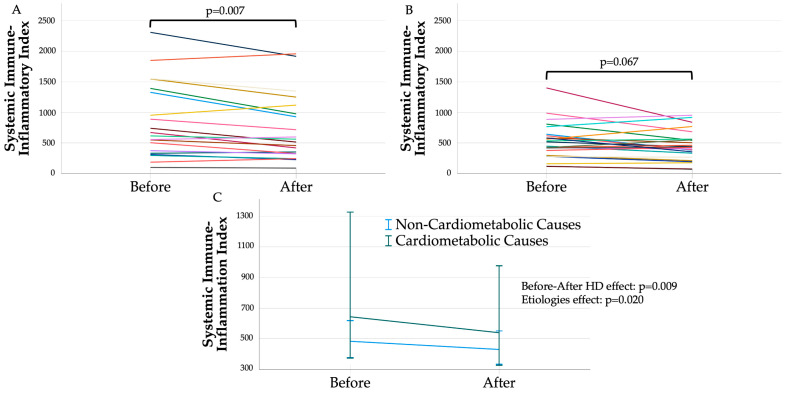
Systemic immune–inflammation index before and after hemodialysis in each patient with cardiometabolic etiology (**A**) and non-cardiometabolic etiology (**B**). (**C**) summarizes before–after effect according to etiologies (values are summarized as median 95%C.I.). Significances were adjusted for age ≥65 years and sex.

Following HD, median SII significantly decreased to 449.1 [342.6–866.6] in the overall population (pre–post HD, *p* = 0.001). Pre–post values of neutrophils, lymphocytes, and platelets are reported in Supplementary Table S2. A significant reduction in SII was observed after HD in patients with cardiometabolic etiologies, but not in those with non-cardiometabolic etiologies. Figure 1 shows individual before–after HD SII values in patients with cardiometabolic etiologies (Panel A, median post-HD SII: 539.1 [324.8–1083.4]; *p* = 0.007) and in those with non-cardiometabolic etiologies (Panel B, median post-HD SII: 429.7 [284.3–560.0]; *p* = 0.067). Panel C illustrates the overall pre–post-HD effect (*p* = 0.009) and the significant impact of etiology (*p* = 0.020) after adjustment for sex and age ≥65 years.

**Figure 2:**
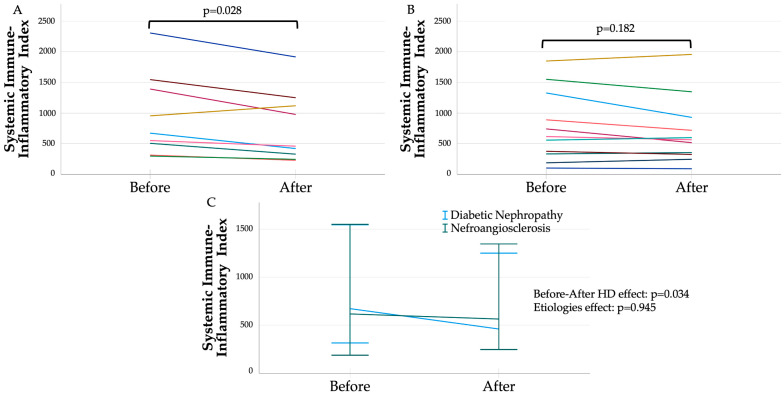
Systemic immune–inflammation index before and after hemodialysis in each patient with diabetic nephropathy etiology (**A**) and nephroangiosclerosis etiology (**B**). (**C**) summarizes before–after effect according to etiologies (values are summarized as median 95%C.I.). Significances were adjusted for age ≥65 years and sex.

An exploratory sub-analysis was conducted in patients with cardiometabolic etiologies (n = 20). A significant reduction in SII was also observed after HD in patients with diabetes (n = 9), but not in those with nephroangiosclerosis (n = 11). Figure 2 presents individual pre–post-HD SII values in patients with diabetes (Panel A, median baseline SII: 671.1 [408.7–1469.1]; median post-HD SII: 458.3 [285.7–1184.4]; *p* = 0.028) and in those with nephroangiosclerosis (Panel B, median post-HD SII: 429.7 [284.3–560.0]; *p* = 0.182). Panel C illustrates the overall pre–post-HD effect (*p* = 0.034) and the effect of etiology (*p* = 0.945) after adjustment for sex and age ≥ 65 years, in patients with cardiometabolic etiologies (n = 20).

To explore the metabolic determinants of inflammatory response, we examined baseline biochemical variables as predictors of SII relative variation. Table 2 summarizes the predictors of SII variations after HD, evaluated as relative change in comparison to baseline. Notably, higher baseline total cholesterol (*p* = 0.001) and GGT (*p* = 0.034) were independently associated with a blunted reduction in SII, indicating that patients with more adverse metabolic profiles exhibited a reduced inflammatory responsiveness to dialysis. Conversely, higher baseline glycaemia (*p* = 0.021) was significantly associated with a greater post-HD reduction in SII, suggesting that elevated glucose levels—possibly reflecting enhanced metabolic activation—may amplify the capacity to acutely modulate immune–inflammatory tone. No significant associations were observed with age, mean blood pressure, or non-cardiometabolic etiology.

## 4. Discussion

This study highlights the pivotal role of metabolic status in modulating the acute inflammatory response to HD. Beyond confirming that HD transiently reduces systemic inflammation—as assessed by the SII—our findings show that this effect is significantly influenced by baseline metabolic parameters, including cholesterol, glycaemia, and gamma-glutamyl transferase (GGT). These results support the concept of a dynamic interplay between metabolic regulation and immune–inflammatory activity in ESRD.

In this observational study, we evaluated the acute effects of a single HD session on systemic inflammation in patients with ESRD, using the SII as a composite biomarker. Our findings demonstrate a significant post-HD reduction in SII in the overall population, particularly in patients with cardiometabolic disease. The post-HD reduction was observed also in the subgroup of patients with diabetes. However, the last finding should be interpreted with caution due to the exploratory nature of the sub-analysis and small simple size of the subgroup. These data suggest that SII may represent a sensitive and dynamic marker of the immuno-inflammatory response to extracorporeal circulation. In this regard, it is important to clarify that the post-dialysis decrease in SII should not be interpreted as an improvement in the chronic inflammatory state of HD patients, which remains well documented and strongly associated with cardiovascular risk [27]. Instead, this reduction more likely reflects acute and transient hematological and immunological shifts occurring during extracorporeal circulation, including neutrophil margination, lymphocyte redistribution, platelet activation/consumption, and partial clearance of circulating cytokines. Similar transient fluctuations have been reported for classical markers such as tumor necrosis α (TNF-α), which significantly decreases after HD and rebounds within 48 h [28]. Moreover, it is known that c-reactive protein (CRP) and interleukin 6 (IL-6) often show only minor peri-dialytic variations [29], supporting the concept that cell-derived indices like SII may provide complementary information on the dynamic immune–inflammatory modulation induced by HD.

The reduction in leukocyte and platelet counts observed after HD is consistent with prior studies suggesting that dialysis may exert transient anti-inflammatory effects via dilutional mechanisms, removal of circulating cytokines, or improved biocompatibility of modern membranes [15,18,19,30]. However, the inflammatory response to HD is multifactorial: mechanical stress, complement activation, oxidative stress, and endothelial dysfunction may all enhance immune activation during treatment, potentially explaining the heterogeneity in SII responses [31,32].

Importantly, our results reveal that the magnitude of the inflammatory modulation is not solely determined by dialysis-related factors or disease etiology but also by individual metabolic status. Subgroup analyses revealed that patients with cardiometabolic comorbidities and diabetes experienced the most significant reductions in SII, possibly reflecting a higher baseline inflammatory burden with greater potential for acute modulation. By contrast, patients with nephroangiosclerosis did not experience significant change in SII, which may reflect a more stable vascular–inflammatory phenotype. This aligns with the hypothesis that vascular aging and stiffening, typical of nephroangiosclerosis, may result in less reactive immune profiles compared to metabolically active conditions such as diabetes or atherosclerosis [9,16].

Importantly, baseline metabolic parameters influenced SII variation: higher levels of total cholesterol and GGT were associated with lower reductions in SII, while elevated glycaemia predicted greater decreases. These associations support the growing recognition of liver function and glucose metabolism as modulators of systemic inflammation in CKD and HD settings [11,21,26].

Multivariate analysis identified baseline total cholesterol and GGT as negative predictors of inflammatory responsiveness, suggesting that altered lipid metabolism and subclinical hepatic dysfunction may dampen the acute anti-inflammatory effects of HD. Conversely, higher glycaemia was associated with greater SII reduction, a counterintuitive but potentially meaningful finding that we speculate may reflect enhanced immune-metabolic activation in hyperglycemic states. These associations support the concept of a metabolic–immune crosstalk in ESRD, where glucose, lipid, and hepatic metabolic pathways modulate not only baseline inflammation but also its short-term dynamics during dialysis.

The utility of SII as an inflammatory biomarker in nephrology is increasingly recognized. Elevated SII has been associated with poor prognosis in cardiovascular disease [33], acute coronary syndromes in CKD [34], urinary albumin excretion [21,22], and mortality in patients with SARS-CoV-2 infection and CKD [35]. However, few studies have examined its acute dynamics during HD. Our study provides the first evidence on a potential anti-inflammatory acute effect of HD, in the context of an end-stage disease characterized by a chronic worsening of the inflammatory state [15,19]. Although we did not repeat the assessment of the SII immediately prior to the subsequent dialysis session, the SII difference observed before–after dialysis strongly suggests a progressive increase in the inflammatory milieu during the inter-dialytic period. This trend may be further influenced by metabolic alterations, including dyslipidemia, insulin resistance, and hepatic stress.

Our study has limitations. The sample size was limited and derived from a single center, which may reduce the generalizability of the findings. Second, we did not include established inflammatory biomarkers such as CRP, IL-6, interleukin 1β (IL-1β), or TNF-α, which prevented direct comparisons between SII dynamics and cytokine responses. This is relevant because previous studies have shown that these markers may remain stable or exhibit only modest changes during dialysis [28], while others, such as TNF-α, can transiently decrease post-HD and rebound within 48 h [29]. Therefore, the decrease in SII observed in our study should be regarded as an acute and likely transient phenomenon rather than as a reflection of overall chronic inflammation, which is consistently present in HD patients [27]. Residual confounders such as nutritional status, medication use, or dialysis membrane type were not systematically analyzed. Finally, the absence of longitudinal follow-up during the inter-dialytic period prevented us from assessing the rebound of SII and its potential prognostic role.

The low cost, broad availability, and integrative nature of SII make it an attractive candidate for routine inflammatory monitoring in dialysis patients and open important further directions. While traditional markers such as CRP or IL-6 provide valuable information, they are more costly, less frequently available, and slower to respond to rapid physiological changes. Should future, larger-scale studies confirm our findings, SII may complement or, in selected contexts, replace these markers for real-time tracking of inflammatory burden. In addition, future studies should explore whether repeated SII assessments can guide therapeutic adjustments (e.g., membrane selection, anticoagulation strategy) or predict clinical outcomes such as infection, hospitalization, or cardiovascular events in the HD population.

Nonetheless, the observed associations between baseline metabolic variables and the degree of inflammatory modulation highlight the relevance of assessing metabolic phenotype when evaluating inflammation in ESRD patients. Future studies should integrate metabolic and inflammatory profiling to identify subgroups more likely to benefit from targeted interventions—whether pharmacologic, dietary, or dialysis-based—to modulate systemic inflammation.

## 5. Conclusions

This study demonstrates that systemic inflammation, as assessed by the SII, is acutely modulated by a single session of HD. The reduction in SII was most pronounced in patients with cardiometabolic disease (−16.2%), suggesting a greater inflammatory plasticity in these populations. The exploratory sub-analysis showed that patients with nephroangiosclerosis showed limited modulation, potentially reflecting a more stable vascular–inflammatory milieu. Crucially, baseline metabolic parameters emerged as independent predictors of the inflammatory response, highlighting the relevance of metabolic health in shaping acute immune dynamics during dialysis. Integrating metabolic and inflammatory biomarkers may represent a promising avenue for precision medicine approaches in nephrology.

## Figures and Tables

**Table 1:** Baseline characteristics according to end-stage renal disease etiology.

	Whole (n = 44)	Non-CM Etiologies (n = 24)	CM Etiologies (n = 20)	*p*
Female (n, %)	14, 31.8%	8, 33.3%	6, 30.0%	0.813
Age (years, mean ± SD)	66.1 ± 13.9	62.0 ± 13.4	71.0 ± 13.2	0.031
Smokers (n, %)	3, 6.8%	2, 8.3%	1, 5.0%	0.662
Diabetes (n, %)	14, 31.8%	3, 12.5%	11, 55.0%	0.003
Hypertension (n, %)	36, 81.8%	16, 66.7%	20, 100%	0.004
CVD (n, %)	22, 55%	10, 41.7%	12, 60.0%	0.226
Blood glucose (mg/dL, mean ± SD)	112.4 ± 25.6	110.5 ± 22.8	114.8 ± 29.9	0.582
SBP (mmHg, median [IQR])	140.0 [130.0–160.0]	132.5 [117.5–155.0]	140.0 [130.0–160.0]	0.114
DBP (mmHg, median [IQR])	70.0 [60.0–80.0]	70.0 [63.5–85.0]	75.0 [64.0–90.0]	0.368
Creatinine (mg/dL, mean ± SD)	8.2 ± 2.4	8.7 ± 2.2	7.6 ± 2.7	0.124
Urea (mg/dL, mean ± SD)	114.4 ± 30.5	116.7 ± 29.9	111.6 ± 31.7	0.582
Total Cholesterol (mg/dL, mean ± SD)	131.1 ± 37.9	126.9 ± 39.1	136.1 ± 36.8	0.428
Triglycerides (mg/dL, median [IQR])	116.0 [71.5–167.5]	104.0 [78.5–140.0]	116.0 [71.5–167.5]	0.671
AST (UI/L, median [IQR])	13.0 [11.5–15.0]	11.5 [10.0–17.5]	13.0 [11.5–15.0]	0.627
ALT (UI/L, median [IQR])	10.0 [5.0–12.5]	8.5 [6.5–15.0]	10.0 [5.0–12.5]	0.705
GGT (UI/L, median [IQR])	22.0 [12.5–30.5]	21.5 [17.0–27.5]	22.0 [12.5–30.5]	0.705
***Etiologies causing end-stage renal disease***				
Connective tissue disease (n, %)	1, 2.3%	1, 4.2%	-	-
Phenylketonuria (n, %)	1, 2.3%	1, 4.2%	-	-
Retroperitoneal fibrosis (n, %)	1, 2.3%	1, 4.2%	-	-
Kidney transplantation failure (n, %)	1, 2.3%	1, 4.2%	-	-
Urosepsis (n, %)	1, 2.3%	1, 4.2%	-	-
Glomerulonephritis (n,%)	4, 9.1%	4, 16.7%	-	-
Diabetic nephropathy (n, %)	15, 34.1%	15, 62.5%	-	-
Nephroangiosclerosis (n,%)	9, 20.5%	-	9, 45.0%	-
Polycystic kidney (n, %)	11, 25.0%	-	11, 55.0%	-

Abbreviations: CM: Cardiometabolic; CVD: cardiovascular disease; IQR: interquartile range; SBP: systolic blood pressure; DBP: diastolic blood pressure; AST: aspartate aminotransferase; ALT: alanine aminotransferase; GGT: gamma-glutamyl transferase.

**Table 2:** Predictors of systemic inflammation index relative variation before–after hemodialysis.

Variable	Mean Change in SII Relative Variation	Standard Error	95% Confidence Interval		*p*
Male gender	4.004	6.381	−8.503	16.512	0.530
NC etiologies	4.657	6.695	−8.466	17.780	0.487
Age (per year)	0.280	0.248	−0.205	0.765	0.258
Total Cholesterol (per 1% increase)	0.262	0.084	0.097	0.428	0.002
GGT (per mg/dL)	0.543	0.259	0.036	1.051	0.036
Blood glucose (per 1 mg/dL)	−0.309	0.137	−0.578	−0.041	0.024
Mean Blood Pressure (per 1 mmHg)	−0.256	0.240	−0.727	0.214	0.286
Basal SII (per 1 mg/dL)	−0.001	0.007	−0.014	0.013	0.901
Time in HD (months)	−0.024	0.040	−0.103	0.056	0.557

Abbreviations: NC: Non-cardiometabolic; GGT: gamma-glutamyl transferase; SII: systemic inflammation index; HD: hemodialysis.

## Data Availability

The datasets presented in this article are not readily available. Requests to access the datasets should be directed to the corresponding author.

## Supplementary Materials

The following supporting information can be downloaded at https://www.mdpi.com/article/10.3390/metabo15100651/s1. Supplementary Table S1. Etiology of end-stage kidney failure; Supplementary Table S2. Univariate correlations between baseline inflammatory systemic index and other continuous variables.

## Abbreviations

The following abbreviations are used in this manuscript:

CVD	Cardiovascular disease
CKD	Chronic kidney disease
ERSD	End-stage renal disease
HD	Hemodialysis
SII	Systemic immune–inflammation index
AST	Aspartate transaminase
ALT	Alanine transaminase
GGT	Gamma-glutamyl transaminase
TNF-α	Tumor necrosis factor α
CRP	C-reactive protein
IL-6	Interleukin 6
IL-1β	Interleukin 1β

## References

[1] Medzhitov R. (2008). Origin and physiological roles of inflammation. Nature.

[2] Sanada F., Taniyama Y., Muratsu J., Otsu R., Shimizu H., Rakugi H., Morishita R. (2018). Source of Chronic Inflammation in Aging. Front. Cardiovasc. Med..

[3] Arnold N., Koenig W. (2025). Inflammation in atherosclerotic cardiovascular disease: From diagnosis to treatment. Eur. J. Clin. Investig..

[4] Liu Y., Guan S., Xu H., Zhang N., Huang M., Liu Z. (2023). Inflammation biomarkers are associated with the incidence of cardiovascular disease: A meta-analysis. Front. Cardiovasc. Med..

[5] Shi L., Wang J., Wei T., Liang Z., Zhang L., Li C., Liu T., Fan W., Zhang M. (2025). Analysis of research trends and hotspots in the primary treatment of end-stage renal disease. Int. Urol. Nephrol..

[6] Dabravolski S.A., Orekhova V.A., Baig M.S., Bezsonov E.E., Starodubova A.V., Popkova T.V., Orekhov A.N. (2021). The Role of Mitochondrial Mutations and Chronic Inflammation in Diabetes. Int. J. Mol. Sci..

[7] Fołta J., Rzepka Z., Wrześniok D. (2025). The Role of Inflammation in Neurodegenerative Diseases: Parkinson’s Disease, Alzheimer’s Disease, and Multiple Sclerosis. Int. J. Mol. Sci..

[8] Miguel V., Shaw I.W., Kramann R. (2025). Metabolism at the crossroads of inflammation and fibrosis in chronic kidney disease. Nat. Rev. Nephrol..

[9] Furman D., Campisi J., Verdin E., Carrera-Bastos P., Targ S., Franceschi C., Ferrucci L., Gilroy D.W., Fasano A., Miller G.W. (2019). Chronic inflammation in the etiology of disease across the life span. Nat. Med..

[10] Grassi D., Desideri G., Ferri C. (2011). Cardiovascular risk and endothelial dysfunction: The preferential route for atherosclerosis. Curr. Pharm. Biotechnol..

[11] Desideri G., Bravi M.C., Tucci M., Croce G., Marinucci M.C., Santucci A., Alesse E., Ferri C. (2003). Angiotensin II inhibits endothelial cell motility through an AT1-dependent oxidant-sensitive decrement of nitric oxide availability. Arter. Thromb. Vasc. Biol..

[12] Kelly D.M., Kelleher E.M., Rothwell P.M. (2025). The Kidney-Immune-Brain Axis: The Role of Inflammation in the Pathogenesis and Treatment of Stroke in Chronic Kidney Disease. Stroke.

[13] Wakamatsu T., Yamamoto S., Yoshida S., Narita I. (2024). Indoxyl Sulfate-Induced Macrophage Toxicity and Therapeutic Strategies in Uremic Atherosclerosis. Toxins.

[14] Waheed Y.A., Liu J., Almayahe S., Sun D. (2025). The role of hyperuricemia in the progression of end-stage kidney disease and its molecular prospective in inflammation and cardiovascular diseases: A general review. Ther. Apher. Dial..

[15] Kadatane S.P., Satariano M., Massey M., Mongan K., Raina R. (2023). The Role of Inflammation in CKD. Cells.

[16] Lauxen J.S., Vondenhoff S., Junho C.V.C., Martin P., Fleig S., Schütt K., Schulze-Späte U., Soehnlein O., Prates-Roma L., Döring Y. (2025). Neutrophil Function in Patients with Chronic Kidney Disease: A Systematic Review and Meta-Analysis. Acta Physiol..

[17] Desideri G., Panichi V., Paoletti S., Grassi D., Bigazzi R., Beati S., Bernabini G., Rosati A., Ferri C., Taddei S. (2011). Soluble CD40 ligand is predictive of combined cardiovascular morbidity and mortality in patients on haemodialysis at a relatively short-term follow-up. Nephrol. Dial. Transpl..

[18] Mihai S., Codrici E., Popescu I.D., Enciu A.M., Albulescu L., Necula L.G., Mambet C., Anton G., Tanase C. (2018). Inflammation-Related Mechanisms in Chronic Kidney Disease Prediction, Progression, and Outcome. J. Immunol. Res..

[19] Friedrich B., Alexander D., Janessa A., Häring H.U., Lang F., Risler T. (2006). Acute effects of hemodialysis on cytokine transcription profiles: Evidence for C-reactive protein-dependency of mediator induction. Kidney Int..

[20] Hu B., Yang X.R., Xu Y., Sun Y.F., Sun C., Guo W., Zhang X., Wang W.M., Qiu S.J., Zhou J. (2014). Systemic immune-inflammation index predicts prognosis of patients after curative resection for hepatocellular carcinoma. Clin. Cancer Res..

[21] Qin Z., Li H., Wang L., Geng J., Yang Q., Su B., Liao R. (2022). Systemic Immune-Inflammation Index Is Associated With Increased Urinary Albumin Excretion: A Population-Based Study. Front. Immunol..

[22] Jin M., Yuan S., Yuan Y., Yi L. (2021). Prognostic and Clinicopathological Significance of the Systemic Immune-Inflammation Index in Patients With Renal Cell Carcinoma: A Meta-Analysis. Front. Oncol..

[23] Wang Q., Zhu S.R., Huang X.P., Liu X.Q., Liu J.B., Tian G. (2021). Prognostic value of systemic immune-inflammation index in patients with urinary system cancers: A meta-analysis. Eur. Rev. Med. Pharmacol. Sci..

[24] Karakaya D., Güngör T., Cakıcı E.K., Yazılıtaş F., Celikkaya E., Bulbul M. (2023). Determining the effectiveness of the immature granulocyte percentage and systemic immune-inflammation index in predicting acute pyelonephritis. Postgrad. Med..

[25] Kocaaslan R., Dilli D., Çitli R. (2024). Diagnostic Value of the Systemic Immune-Inflammation Index in Newborns with Urinary Tract Infection. Am. J. Perinatol..

[26] Lai W., Xie Y., Zhao X., Xu X., Yu S., Lu H., Huang H., Li Q., Xu J.Y., Liu J. (2023). Elevated systemic immune inflammation level increases the risk of total and cause-specific mortality among patients with chronic kidney disease: A large multi-center longitudinal study. Inflamm. Res..

[27] Wang Y., Gao L. (2022). Inflammation and Cardiovascular Disease Associated With Hemodialysis for End-Stage Renal Disease. Front. Pharmacol..

[28] Guerrero F., Carmona A., Jiménez M.J., Ariza F., Obrero T., Berdud I., Carrillo-Carrión C., Rodríguez M., Soriano S., Muñoz-Castañeda J.R. (2025). New biomarkers of inflammation associated with haemodialysis. Clin. Kidney J..

[29] Dheda S., Vesey D.A., Hawley C., Johnson D.W., Fahim M. (2022). Effect of a Hemodialysis Session on Markers of Inflammation and Endotoxin. Int. J. Inflam..

[30] Cobo G., Lindholm B., Stenvinkel P. (2018). Chronic inflammation in end-stage renal disease and dialysis. Nephrol. Dial. Transpl..

[31] Tarakçioğlu M., Erbağci A.B., Usalan C., Deveci R., Kocabaş R. (2003). Acute effect of hemodialysis on serum levels of the proinflammatory cytokines. Mediat. Inflamm..

[32] Caglar K., Peng Y., Pupim L.B., Flakoll P.J., Levenhagen D., Hakim R.M., Ikizler T.A. (2002). Inflammatory signals associated with hemodialysis. Kidney Int..

[33] Ye Z., Hu T., Wang J., Xiao R., Liao X., Liu M., Sun Z. (2022). Systemic immune-inflammation index as a potential biomarker of cardiovascular diseases: A systematic review and meta-analysis. Front. Cardiovasc. Med..

[34] Shi S., Kong S., Ni W., Lu Y., Li J., Huang Y., Chen J., Lin K., Li Y., Ke J. (2023). Association of the Systemic Immune-Inflammation Index with Outcomes in Acute Coronary Syndrome Patients with Chronic Kidney Disease. J. Inflamm. Res..

[35] Ozdemir A., Kocak S.Y., Karabela S.N., Yılmaz M. (2022). Can systemic immune inflammation index at admission predict in-hospital mortality in chronic kidney disease patients with SARS-CoV-2 infection?. Nefrología.

