# Supplementary material for: Metabolic Determinants of Systemic Inflammation Dynamics During Hemodialysis: Insights from the Systemic Immune–Inflammation Index in a Single-Center Observational Study
Source: Metabolites. 2025 Sep 30;15(10):651. doi: 10.3390/metabo15100651 (PMC12566289; doi:10.3390/metabo15100651)
Supplement: Supplementary file 1 [file metabolites-15-00651-s001.zip › metabolites-3792485-supplementary.pdf]

Supplementary Table S1. Univariate correlations between baseline inflammatory systemic index and other continuous variables.

		Baseline Inflammatory systemic Index
Age	Rho di Spearman.	-0.139
	P.	0.368
	N.	44
Systolic BP	Rho di Spearman.	-0.255
	P.	0.095
	N.	44
Diastolic BP	Rho di Spearman.	-0.142
	P.	0.356
	N.	44
Glycaemia	Rho di Spearman.	0.127
	p.	0.412
	n.	44
Urea	Rho di Spearman.	0.022
	p.	0.888
	n.	44
Creatinine	Rho di Spearman.	0.144
	p.	0.349
	n.	44
Total Cholesterol	Rho di Spearman.	-0.101
	p.	0.515
	n.	44
Triglycerides	Rho di Spearman.	0.039
	p.	0.800
	n.	44
AST	Rho di Spearman.	-0.053
	p.	0.734
	n.	44
ALT	Rho di Spearman.	-0.045
	p.	0.773
	n.	44
GGT	Rho di Spearman.	-0.091
	p.	0.555
	n.	44

Supplementary Table S2. Neutrophils, lymphocytes and platelets before and after haemodialysis (HD)

	Before HD	After (HD)	p
Neutrophils ($x10^9/l$)	3.9 [2.9-4.8]	3.2 [2.6-4.6]	<0.001
Lymphocytes ($x10^9/l$)	1.3 [0.9-1.5]	1.1 [0.8-1.4]	0.002
Platelets ($x10^9/l$)	170.5 [146.5-220.5]	167.0 [119.7-208.5]	0.002
